# A 3D printed esophageal atresia–tracheoesophageal fistula thorascopy simulator for young surgeons

**DOI:** 10.1038/s41598-024-62154-4

**Published:** 2024-05-20

**Authors:** Joong Kee Youn, Dayoung Ko, Hee-Beom Yang, Hyun-Young Kim

**Affiliations:** 1 Department of Pediatric Surgery, Seoul National University Hospital, Seoul, Korea; 2 Department of Pediatric Surgery, Seoul National University College of Medicine, 101 Daehak-ro, Jongro-gu, Seoul, 03080 Korea; 3 Department of Surgery, Seoul National University Bundang Hospital, Seongnam, Korea

**Keywords:** 3D printing, Esophageal atresia, Professional education, Paediatric research, Gastrointestinal models

## Abstract

We developed a 3D-printed thoracoscopic surgery simulator for esophageal atresia with tracheoesophageal fistula (EA-TEF) and assessed its effectiveness in educating young pediatric surgeons. Prototype production and modifications were repeated five times before producing the 3-D printed final product based on a patient’s preoperative chest computed tomography. A 24-item survey was used to rate the simulator, adapted from a previous report, with 16 young surgeons with an average of 6.2 years of experience in pediatric surgery for validation. Reusable parts of the thoracic cage were printed to combine with replaceable parts. Each structure was fabricated using diverse printing materials, and subsequently affixed to a frame. In evaluating the simulator, the scores for each factor were 4.33, 4.33, 4.27, 4.31, 4.63, and 4.75 out of 5, respectively, with the highest ratings in value and relevance. The global rating was 3.38 out of 4, with ten stating that it could be used with slight improvements. The most common comment from participants was that the esophageal anastomosis was close to the actual EA-TEF surgery. The 3D-printed thoracoscopic EA-TEF surgery simulator was developed and reflected the actual surgical environment. It could become an effective method of training young pediatric surgeons.

## Introduction

In surgical education, young trainees have acted as apprentices, slowly gaining knowledge and understanding of procedures from senior surgeons^1^. The apprentice model has been favored because it promotes the exposure of surgical residents to the passion and commitment that surgeons have to their patients and practice^1^. One significant limitation is the absence of well-organized programs that specifically teach essential skills^2,3^. Also, in medical education, there is less focus on rare disorders^4^, and due to ethical reasons, teaching surgery with animal models has been replaced by realistic simulations^5^.

Achieving thoracoscopic correction of esophageal atresia with a tracheoesophageal fistula (EA-TEF) necessitates substantial expertise in neonatal minimally invasive surgical (MIS) techniques^6^. The unique technical features include a very small workspace and fragile tissue^7^. The rarity of these neonatal MIS procedures has led to a correspondingly steeper learning curve for young and novice pediatric surgeons and residents^8,9^. Due to these conditions, sufficient training cannot be achieved in clinical practice alone^10^. Research has demonstrated the effectiveness of simulation-based training in enhancing operating room performance, leading to reduced operating time and a lower incidence of intraoperative errors^11^. Simultaneously, simulation-based training holds significant potential for enhancing the standard of surgical care for neonates undergoing complex procedures like EA-TEF repair^12^.

Recently, EA-TEF surgery training tools have been developed. Barsness et al.^13,14^ developed rapid-prototyped hybrid-type or synthetic simulators for EA-TEF repair, while Wells et al. reported serial development of synthetic simulators, removing ethical issues of using animal intestines^17–19^. However, more realistic use of materials and a need for education on the entire process of EA-TEF repair, including trocar insertion, have led us to develop this 3D-printing-based thoracoscopic surgical simulator derived from computed tomography (CT) of an actual EA-TEF patient.

Thus, we developed a 3D-printed thoracoscopic surgery simulator for EA-TEF surgery and checked its effectiveness in the education of young pediatric surgeons.

## Methods

### Validation process

#### Validation procedure

Before validation, internal developers conducted experimental trial runs to incorporate final modifications and initiate the validation process. The validation participants were members of the Korean Society of Pediatric Surgery. Since it was designed to educate young surgeons, we emailed the members to recruit volunteers with < 10 years of specialist experience.

Validation and surveys were conducted in two sessions, chosen at times convenient for the Medtronic Innovation Center and our center’s operating room. Subsequently, evaluations and survey items were investigated to obtain feedback on the simulator.

#### Survey component

A 24-item survey was used to rate the simulator and consisted of 23 5-point rating scales (physical attributes, realism of materials, realism of experience, ability to perform tasks, value, and relevance) and one 4-point global rating scale with free comments for improvements adapted from Barsness et al.^14^.

#### Participant Information

There were 16 participants, with a median surgical experience of 6.2 years (range 0.2–13) for general surgeons (Table 1). Among them, eight (50%) were pediatric surgery specialists. The median experience in EA surgery leadership was two times, assisting in ten cases, and among them, the median experience in minimally invasive EA surgery leadership was once with assisting four times.

**Table 1:** Demographics of participants in the validation.

Items	N = 16
Experience as a surgeon (median, years)	6.2 (0.2–13)
Certified pediatric surgeon (%)	8 (50.0)
Experience as a pediatric surgeon (median, years)	3.5 (0–7)
Experiences in performing EA-TEF surgery (median)	2 (0–10)
Experiences in assisting EA-TEF surgery (median)	10 (1–30)
Experiences in performing thoracoscpic EA-TEF surgery (median)	1 (0–10)
Experiences in assisting thoracoscpic EA-TEF surgery (median)	4 (0–30)

### Simulator development process

The simulator development process proceeded as follows. We selected a patient's chest CT scan as the basis for 3D reconstruction and implemented it. We discussed the implementation of structural elements in the simulator and printing materials. Printing was carried out, the fixed and replaceable parts were identified, and developers used it experimentally after undergoing candidate adjustments such as structural and printing material modifications. After the final printing, a validation process was started, targeting pediatric surgeons, and the results were analyzed along with survey responses.

#### Patient selection

A patient who underwent surgery for EA-TEF at our institution and had undergone a chest CT scan before surgery was selected and the DICOM files were extracted. The chosen patient was a male born in another hospital at gestational age 38 + 0 weeks, weighing 2280 g, who underwent a chest CT on the day after birth for evaluation before transfer to our institution. This hospital had limited experience in diagnosing and treating EA patients, so they struggled with diagnosing and even went as far as conducting a CT scan before transferring them to our institution. The DICOM files of this patient's CT were sent to a 3D printing agency for primary 3D reconstruction.

#### Pre-production stage

The 3D reconstruction was created to be visible in images, and based on this, the basic plan for 3D printing was established. Fixed and replaceable parts were initially separated for printing. The fixed parts included the ribs and sternum, which comprised the thorax cavity, with a replaceable skin covering the ribs. Nipples were marked as landmarks. Replaceable parts included the proximal pouch of the esophagus, distal esophagus, trachea, azygous vein, inferior vena cava (IVC), right atrium, and vagus nerve. Materials for these were determined, mainly focusing on Agilus30, Agilus95, and silicon, considering the properties of the organs that needed to be created through trial and error.

#### 3D printing process

Preoperative CT imaging with 3 mm slices was sent to Medical IP©, a 3D printing agency. Medical IP© uses the MEDIP software platform (http://medicalip.com/Medip, MEDICAL IP©, Seoul, Republic of Korea) for medical image processing, which applies machine learning-based thresholds, region growing, and graph-cut algorithms. To reconstruct CT images in three dimensions, the segmentation of each organ is performed and reviewed by clinicians.

The 3D printing model was manufactured on a 1:1 scale and printed with Agilus30 (100 g), Agilus95 (350 g), and silicon (200 g) using the Polyjet method (J750 [Stratasys; 7665 Commerce Way Eden Prairie, MN 55344, USA]). The simulator was printed separately for each part and then assembled together to complete the simulator.

#### Post-printing

After creating the initial simulator prototype, five rounds of modifications were made. During the modification process, anatomical structure modifications were carried out, primarily involving continuous fine-tuning of materials to make them more realistic. Lifts were added to adjust the simulator's height and direction based on the position of the examiner when the material was complete.

### Configuration of the developed simulator

**Figure 1:**
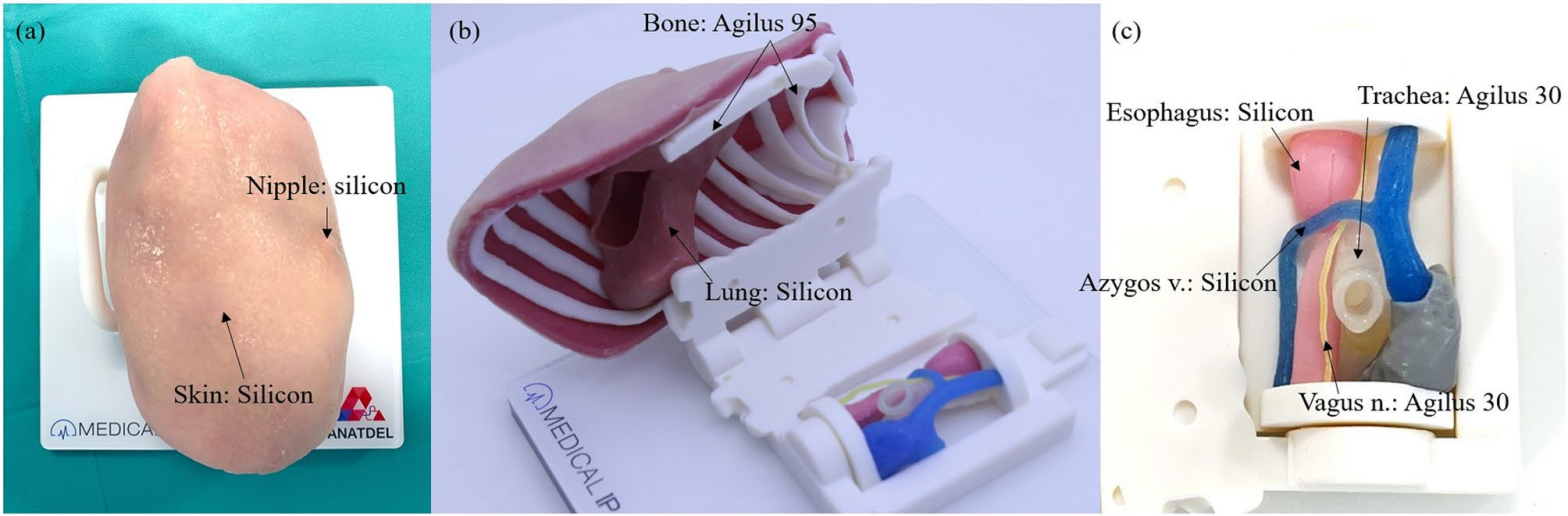
Details of the simulator with printed intrathoracic organs and materials.

As planned, the simulator was designed with distinct fixed and replaceable components (Fig. 1). The fixed portion was constructed on a plastic plate, consisting of the thorax with rib cage, sternum, and scapula, made from Agilus95 for bones (Fig. 1a,b).

The replaceable portion was designed as a cassette, allowing insertion and removal, and it was composed of organs for actual surgical practice (Fig. 1c). The replaceable part was secured using magnets to ensure that it remained stationary. The proximal and distal portions of the esophagus were 3D printed layer by layer using silicone, while the trachea was printed using Agilus30. The azygous vein was connected to the superior vena cava (SVC), extending to the right atrium, and printed in silicone to express elasticity during ligation. After completing the entire surgical procedure, the replaceable parts could be removed and exchanged for repeated attempts at the surgery.

The thoracic cage was equipped with a handle for opening and closing, and when opened, it showed a lung made of silicone attached to the ribs in a deflated state, providing a realistic view (Fig. 1b). The thoracic cage was covered with a three-layer printed skin, and the scapula and nipples were marked for palpation, serving as landmarks for trocar insertion during thoracoscopic EA-TEF surgery.

Additionally, to simulate the adjustment of the patient's position in the actual operating room bed when using this simulator, it was equipped with a lift to adjust its height and direction.

This study conformed to the ethical guidelines of the 1975 Helsinki Declaration and was approved by the Institutional Review Board of Seoul National University Hospital (IRB No. 2110-047-1261). Informed consents were obtained from all participants of this study.

## Results

### Evaluation results

Sixteen participants used the simulator for validation. They evaluated it with scores of 4.33, 4.33, 4.27, 4.31, 4.63, and 4.75 out of 5 in the categories of physical attributes, realism of material, realism of experience, ability to perform tasks, value of the simulator, and relevance of simulator to practice, respectively (Table 2). In the physical attributes’ category, chest circumference and scale of the tissue scored the highest at 4.44, while the scapula's landmark tactility scored the lowest at 4.13. The realism of the material had a reasonably even score distribution overall, and in the realism of experience category, high scores were given for anatomy and fistula location. However, the pressure required when inserting the trocar received a lower score in this category. In the ability to perform tasks category, as expected, the evaluator's ability to perform an esophageal anastomosis was the lowest, with ratings in the 3-point range. Regarding value, this simulator received a high rating of 4.81 as a training tool and a good rating of 4.44 as a testing tool.

**Table 2:** Results of validity assessment.

Domain	Observed averages
1. Physical attributes	4.33
Chest circumference	4.44
Chest depth	4.38
Intercostal space	4.38
Landmark tactility, scapula	4.13
Landmark visualization, scapula	4.25
Scale of tissue within the model	4.44
2. Realism of materials	4.33
Overall impression of simulator	4.38
Skin	4.38
Ribs	4.25
Esophagus	4.38
3. Realism of experience	4.27
Amount of pressure needed to place trocars	3.81
Realism of anatomy during repair	4.50
Realism of location of fistula	4.50
Realism of upper pouch anatomy	4.25
4. Rate your ability to perform the below tasks on the simulator	4.31
Acquisition of target trocar sites	4.50
Proper placement of trocars	4.38
Fistula closure	4.31
Opening of upper pouch	4.44
Esophageal anastomosis	3.94
5. Rate the value of the simulator	4.63
Please rate the value of the simulator as a training tool	4.81
Please rate the value of the simulator as a testing tool	4.44
6. Please rate the relevance of this simulator to your practice	4.75

In the global rating, which assesses the overall impression, ten participants indicated that the simulator could be used for training but would benefit from some improvements, while six participants assessed it as suitable for use as a training simulator without the need for improvement (3.38/4 points).

### Survey results

In the free comments received, participants praised the simulator for its overall simulation view being very similar to actual thoracoscopic EA-TEF repair and its similarity to the real experience during esophageal anastomosis. Suggestions for improvement included addressing the issue of rib breakage during trocar insertion due to the rigidity of the thoracic cage and enhancing the soft tissue around the upper and lower esophageal pouches to provide a more realistic dissection experience.

## Discussion

This simulator was constructed based on the CT scans of an actual patient, accurately reflecting the size of the thoracic cavity and organs. Various materials were used to produce each organ with properties as similar as possible, with particular emphasis on creating a sensation similar to esophageal anastomosis, which was validated and confirmed to be valuable. Compared to previously developed simulators, a distinctive feature is the layer-by-layer 3D printing of skin and muscles, enabling trocar insertion in situations resembling actual surgery using skin landmarks. It received ratings of 4 or higher in all domains, and pediatric surgeons evaluated it as valuable as a training tool with some modifications.

Throughout the development process, we meticulously incorporated feedback from developers and test users. Through a series of five prototype iterations during this iterative process, we successfully developed the final version of the simulator. This provides an opportunity for trainee doctors, who may find it challenging to encounter many cases of EA-TEF and cannot directly perform surgeries, to undergo comprehensive and safe skill training.

### Characteristics and differences from previous studies in this research

The validation results of this study applied the criteria presented by Barsness et al.^14^, encompassing six domains. Compared to the 2020 study by Wells et al. and Barsness's hybrid and synthetic model regarding the EA simulator, it cannot be definitively stated whether our simulator is superior or inferior. However, based on these validation results, it can be said that a simulator comparable to them has been developed.

In terms of physical attributes, there were minimal differences compared to previous studies. Notably, items directly derived from actual patient CT scans, such as chest circumference and depth, scored higher. The “Realism of materials” domain scored 4.33, comparable results from Wells and Barsness studies that used synthetic materials. Particularly, the evaluation of the esophagus, a critical component, demonstrated higher realism than the other two studies, indicating increased realism in anastomosis practice. The “Realism of experience” domain, while slightly lower than the hybrid model, outperformed other studies using synthetic materials. In the “Ability to perform tasks” domain, our model scored higher across all items than other synthetic simulator studies. Esophageal anastomosis scored 3.94, surpassing Wells' Model 3 and even the hybrid model, indicating a level of realism close to bovine tissue but achieved with fully synthetic materials.

Our model, capable of executing the entire thoracoscopic EA-TEF surgery process from trocar insertion to azygos vein ligation, fistula ligation, proximal pouch opening, and esophageal anastomosis, stands out as the first simulator with such capabilities. In contrast, Model 3 reflected the anastomosis process but omitted steps like fistula ligation and trocar insertion. Another simulator from a Japanese group focused on intracorporeal suturing and knot tying, excluding steps like fistula ligation and vein ligation^7^. Consequently, when measuring Barsness scores, our model was comparable in all domains compared to previous studies. Particularly, the high realism of the esophagus, coupled with manageable difficulty, suggests that this standard model could provide an opportunity for trainees or junior doctors with limited surgical experience to gain practical experience in the entire surgical process before actual procedures.

### Comparison with other low-cost models

Due to the necessity for an EA-TEF simulator, various low-cost simulator models have been researched and reported^10,15,16^. Maricic et al. constructed a simulator costing around $50, using wood and a plastic tube for the thoracic cage, and created internal structures with latex balloons^15^. Regarding anatomical characteristics and surgical anatomy, 40–50% of respondents viewed these characteristics favorably. In another study published in 2021, two balloons were connected to a suture pad to create a low-cost model, receiving evaluation scores of between 3.7 and 4.1^10^. A model utilizing foley catheters and feeding tubes on a chicken leg, costing 41 euros, was also evaluated as feasible for training^16^.

The limitations of these models may include limited realism in the visual aspects or the ability to implement only anastomosis. However, considering that the cage production for our model costs around $1500 and replaceable parts are approximately $50, these low-cost models could be applied to less experienced physicians before implementing 3D printing-based simulators.

### Limitations

One limitation of this study is the small number of participants with little experience and self-assessment. It is anticipated that further improvements could be identified if validations were conducted by surgeons with diverse experiences, as done in other studies presented at international conferences. Secondly, while the major structures were created to mimic the real environment, the difficulty in simulating soft tissues, such as the fat in these structures, may be noted. This limitation resulted in the inability to implement procedures like proximal pouch dissection.

## Conclusion

Utilizing CT scans from actual EA-TEF patients, we developed a simulator based on 3D printing with entirely synthetic materials to simulate the entire process of thoracoscopic EA-TEF surgery and conducted a validation process. Users evaluated its potential highly as a training tool. If employed in training less experienced pediatric surgeons, we anticipate that this simulator, devoid of ethical concerns, could contribute to overcoming the learning curve.

## Data Availability

The data underlying this article cannot be shared publicly for the privacy of individuals that participated in the study. The data will be shared on reasonable request to the senior author H-YK.
